# Juxtarenal Modular Aortic Stent Graft Infection Caused by* Staphylococcus aureus*

**DOI:** 10.1155/2016/7597265

**Published:** 2016-01-24

**Authors:** Róbert Novotný, Petr Mitáš, Jaroslav Hlubocký, Ján Hrubý, Andrey Slautin, Rudolf Špunda, Jaroslav Lindner

**Affiliations:** 1-2nd Department of Cardiovascular Surgery, General Teaching Hospital and 1st Faculty of Medicine, Charles University, 12000 Prague, Czech Republic

## Abstract

*Introduction*. We are presenting a case report of an infected modular abdominal stent graft.* Case Presentation*. A 67-year-old male patient three years after Cook's modular abdominal aortic aneurysm (AAA) graft implantation for juxtarenal AAA with an implantation of a stent extension into the right common iliac artery for type Ib endoleak. The patient was admitted into our center in severe condition with suspected retroperitoneal bleeding. Computed tomography angiography (CTAG) confirmed retroperitoneal bleeding in the right common iliac artery. An urgent surgical revision was indicated; destructed arterial wall around the stent extension in the right common iliac artery was discovered. Due to the severe state of health of the patient, a resection of the infected stent and affected arterial wall was performed, followed by an iliac-femoral crossover bypass. The patient was transported to the intensive care unit with hepatic and renal failure, with maximal catecholamine support. Combined antibiotic treatment was started. The patient died five hours after the procedure. The cause of death was multiorgan failure caused by sepsis. Hemocultures and perioperative microbiological cultures showed the infection agent to be* Staphylococcus aureus* methicillin sensitive.* Conclusion*. Stent graft infection is a rare complication. Treatment is associated with high mortality and morbidity.

## 1. Introduction

Endovascular abdominal aortic aneurysm repair (EVAR) is an alternative treatment to an open surgical repair of abdominal aortic aneurysm (AAA) [1]. Endoleak is a frequent complication during EAVR. Most endoleaks can be periprocedurally treated with simple endovascular means [2]. Stent graft infection is another serious complication of EAVR. There is insufficient published data to establish the true incidence of post-EVAR infections. However, the incidence of EVAR infection is estimated to be somewhere between 0.6 and 3% [3, 4]. We are presenting a case of a high risk 67-year-old male patient with infected Cook's modular AAA graft and stent extensions in the right common iliac artery (WALLSTENT*™*).

## 2. Case Presentation

**Figure 1:**
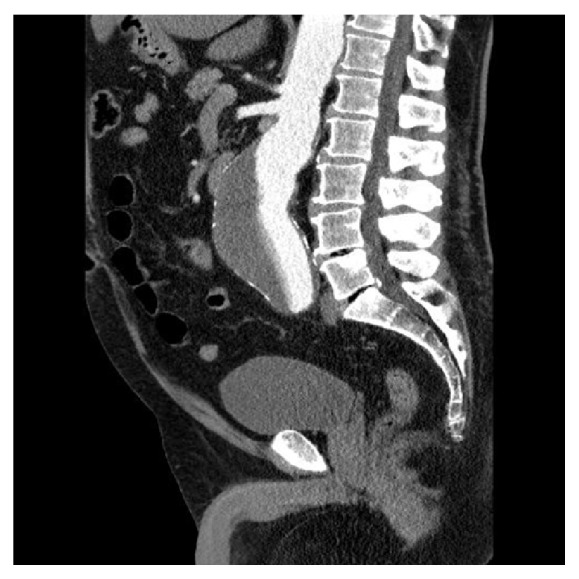
Juxtarenal abdominal aortic aneurysm 66 × 150 mm.

This is a report of a high risk 67-year-old male patient with coronary heart disease (postmyocardial infarction caused by right coronary artery occlusion) with left ventricular ejection fraction 40%, type 2 diabetes mellitus, renal insufficiency (KDOQI: stage 3a), chronic obstructive pulmonary disease (GOLD Criteria: stage 2), and arterial hypertension (WHO ll.). During general medical examination at a different center a pulsating sensation in the abdomen was observed. Computed tomography angiography (CTA) was performed as there was a high suspicion of AAA. Based on the CTA results, the patient was diagnosed with 66 mm × 150 mm juxtarenal AAA (Figure 1). Due to the size of the AAA and the patient's severe comorbidities, EVAR was indicated for the patient. The patient was referred to our center for stent graft implantation. Cook's (Zenith Flex® AAA Endovascular Graft Bifurcated Main Body Graft) bifurcated stent graft was implanted, followed by implantation of a bare metal vascular stent extension into the right common iliac artery (WALLSTENT Endoprosthesis, Boston Scientific) for type Ib endoleak. The procedure was without any complications and the patient recovered and was discharged five days later.

**Figure 2:**
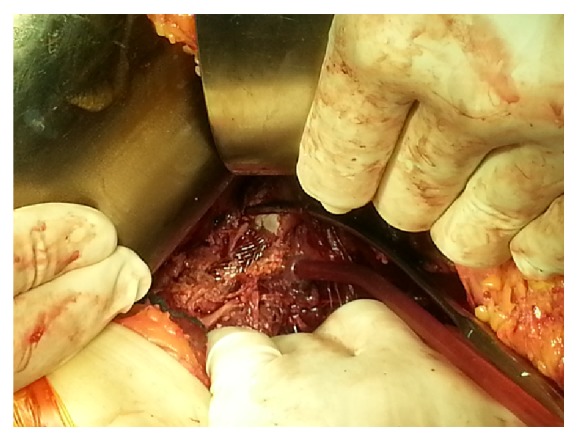
Perioperative finding: destructed wall of a right common iliac artery around bare metal vascular stent.

Three years later, the patient was admitted into a different center for a four-day lasting fever (C-reactive protein: 87, Leukocytes: 12.3 × 10.8 × 10^9^/L, and Hemoglobin: 92 g/L). A broad spectrum antibiotic treatment with intravenous ampicillin was started, as clinicians could not find any source of infection. Due to the deteriorating medical condition, the patient was referred to our center. On admission, the patient was in a severe condition with sepsis (C-reactive protein: 342, Leukocytes: 21 × 10.8 × 10^9^/L, and Hemoglobin: 68 g/L). Hemocultures were taken. CTA was performed, confirming retroperitoneal bleeding in the region of the right common iliac artery. An urgent surgical revision was indicated. Because the pathogen was still undetected, the patient was given a combined antibiotic treatment consisting of gentamycin, Vancomycin, and metronidazole before the surgical procedure. The procedure was performed in a supine position with ipsilateral hip elevation under general anesthesia. Retroperitoneal approach was chosen in order to gain the best access to the right iliac artery. An oblique ski incision form the lateral border of the rectus muscle extending to the midaxillary line halfway between the subcostal margin and iliac crest was performed. The external oblique and internal oblique muscles were divided, and transversus abdominis and transversalis fascia were opened, gaining access to the retroperitoneal space. The peritoneum was fully stripped from the lateral pelvic wall and retracted medially as iliac vessels were exposed. In the retroperitoneum, a massive hematoma was present. As the retroperitoneum was opened, we found complete destruction of 2 cm long of the arterial wall of the right common iliac artery with unmasked bare metal stent (Figure 2). A vascular clamp was placed above and below the arterial defect. Microbiological cultures were taken from the artery, stent, and surrounding tissues. The infected metal stent and affected arterial wall were resected with thorough debridement of the surrounding soft tissues. The proximal part of the artery above the defect was ligated and sutured with Prolene 4/0 suture. In order to prevent ischemia of the right lower extremity, extra-anatomical vascular reconstruction was performed: left to right external iliac-external iliac crossover bypass (THE ADVANTA*™* SST GRAFT 7 mm, Atrium) soaked in rifampicin. The procedure was accompanied by excessive bleeding which was continuously substituted throughout the procedure (total periprocedural blood loss: 4500 mL). The patient was transported into the intensive care unit with catecholamine support 3 *μ*g/kg/min, where a combined antibiotic treatment with Vancomycin and Tienam was started. Five hours after the procedure the patient died of hepatic and renal failure with total metabolic collapse and sepsis (C-reactive protein: 420, Leukocytes: 29 × 10.8 × 10^9^/L, Hemoglobin: 82 g/L, pCO_2_: 6, 08, and lactate: 13.2 mmol/L). Hemocultures and perioperative microbiological cultures showed massive contamination caused by infectious agent* Staphylococcus aureus* methicillin sensitive.

## 3. Discussion

Preoperative evaluation of treatment choices for a patient for whom AAA repair was indicated is crucial in order to reduce not only perioperative risk but also late postprocedural events [5]. Despite the cost-effectiveness ratio, EVAR offers unquestionable benefits in comparison to an open AAA repair in high risk patients that would require an extensive open surgical procedure [6, 7]. Contrary to open AAA repairs, EVAR carries a unique and specific set of complications. The most typical periprocedural and postprocedural complication is endoleak, which can be treated with simple endovascular means [2]. Other complications include graft thrombosis and migration [8]. The incidence of EVAR infection is estimated to be somewhere between 0.6 and 3%, even though there is insufficient data to establish the true incidence of post-EVAR infection [3, 4]. The published data is limited to case reports and series [9]. The most probable sources of infection are thought to be nosocomial infections, perioperative contamination, blood stream septicemia, and surgical site infection.

Stent graft infection poses a severe complication with mortality ranging between 11 and 28% especially in patients with blood stream septicemia [10, 11]. Ducasse et al. reviewed 65 cases of infected stent grafts discovering that the most frequent pathogen causing 55% of infections is* Staphylococcus aureus* [12]. Stent graft infection is generally presented as either severe systemic sepsis, low-grade sepsis, or aortoenteric fistula. Diagnosis relies mainly on suspicion, imaging of the surrounding tissues, and bacteriological cultures [13]. The treatment of an infected stent graft poses a problematic challenge. The main principles of infected stent graft treatment are comparable to those of an infected prosthetic vascular graft: tailored-antibiotic treatment, infected graft explanation followed by anatomical or extra-anatomical revascularization [14]. A partial removal of infected stent graft was reported by Hart et al. in a series of 15 cases with a mortality rate of 27%, thus showing no significant decrease in patient's mortality with the use of this approach [15]. Prophylactic antibiotic admission still remains a greatly discussed topic; however new emerging evidence is suggesting its use for selected cases such as patients with immune deficiency, difficult procedures, prolonged indwelling catheter, high risk of infection, and known colonization of the patient. The adequate length of prophylactic antibiotic treatment is still unknown and further retrospective analysis of published data is needed in order to answer key questions in stent graft infection treatment [9, 16].

## 4. Conclusion

In recent years we have noticed a dramatic increase of EVAR used for AAA. Even though stent graft infections are a rare complication, their treatments are associated with high mortality and morbidity. The exact pathophysiology behind stent graft infection in humans remains unclear. Periprocedural sterility and the use of prophylactic antibiotics in selected patients should be implemented as a gold standard when EVAR is considered. Due to the rarity of stent graft infections, no therapeutic algorithm has been established yet.
